# Potential factors contributing to observed sex differences in virtual-reality-induced sickness

**DOI:** 10.1007/s00221-023-06760-0

**Published:** 2024-01-03

**Authors:** Grainne M. Bannigan, Alexandra A. de Sousa, Meike Scheller, Daniel J. Finnegan, Michael J. Proulx

**Affiliations:** 1 Department of Psychology, University of Bath, Bath, UK; 2 School of Public Health Physiotherapy and Sports Science, University College Dublin, Dublin, Ireland; 3 School of Sciences, Bath Spa University, Bath, UK; 4 School of Psychology, University of Durham, Durham, UK; 5 School of Computer Science and Informatics, Cardiff University, Cardiff, UK; 6 Department of Computer Science, REVEAL Research Centre, University of Bath, Bath, UK

**Keywords:** Virtual reality, Motion sickness, Menstrual cycle, Psychophysiology

## Abstract

Virtual reality (VR) technology has been widely adopted for several professional and recreational applications. Despite rapid innovation in hardware and software, one of the long prevailing issues for end users of VR is the experience of VR sickness. Females experience stronger VR sickness compared to males, and previous research has linked susceptibility to VR sickness to the menstrual cycle (Munafo et al., Exp Brain Res 235(3):889–901). Here we investigated the female versus male experience in VR sickness while playing an immersive VR game, comparing days of the menstrual cycle when hormones peak: day 15 (ovulation—peak estrogen) and day 22 (mid-luteal phase—peak progesterone). We found that immersion duration was greater in the second session than the first, and discomfort was lessened, suggesting a powerful adaptation with repeated exposure. Due to the estrogen levels changing along with the exposure, there was no clear independent impact of that; note, though, that there was a significant difference between self-report and physiological measures implying that GSR is potentially an unreliable measure of motion sickness. Although prior work found a delay over 2 days between session would not allow adaptation and habituation to reduce VR sickness susceptibility, we found that a week delay has potential success.

## Introduction

The experience of motion sickness (MS) in virtual reality (VR), also known as “VR sickness”, suggests significant sex differences with females reporting to suffer VR sickness more frequently than males. In entertainment, experiencing VR sickness may result in an unpleasant experience and impact commercial enterprises because of reduced user retention. In professional applications, for example simulation training, the concerns are more severe: failing to adapt effective interventions for avoiding symptoms of VR sickness may make VR more dangerous for females, creating a barrier for inclusivity and impacting their ability to train in VR. To develop effective future interventions, it is critical that we understand the factors contributing to sex differences and the greater susceptibility to VR sickness in females.

Previous research has attempted to reduce VR sickness by reducing the field of view (Fernandes and Feiner 2016; Al Zayer et al. 2019), adapting to the environment through repeated exposure (Hill and Howarth 2000), and by reducing the latency of the VR system when navigating virtual environments (Meehan et al. 2003). While effective across both sexes, none of these interventions have shown the same level of improvement in females compared to males. Moreover, they do not tell us *why* differences between sexes occur (Hemmerich et al. 2019). Research directly comparing female and male susceptibility to VR sickness (susceptibility to Motion Sickness or MS hereafter) is often neglected, and for understandable reasons as it is difficult. One exception is research showing that MS in females may be influenced by variations in hormone levels which occur throughout the menstrual cycle, specifically estrogen (Clemes and Howarth 2005). While this provides an explainable hypothesis regarding sex differences, more empirical evidence is needed to draw conclusions on whether MS is affected by the menstrual cycle. Most studies regarding VR sickness involve self-reported measures where participants rate their discomfort on a scale in response to questions regarding their experience (Chen et al. 2015; Clemes and Howarth 2005; Young et al. 2006). However, there are known issues with self-report measures: participants may be primed to a certain response, arbitrary units of measurement may be used introducing large variation in responses, and the subjective nature of responses makes individual differences difficult to identify. Objective measures provide less variation in response measurement, making comparisons more reliable. Unfortunately, beyond observable sickness such as vomiting, there is no agreement across the field on what such objective measures might be such that the subjective were rendered unnecessary.

As noted, this topic is understudied because such designs are challenging as the menstrual cycle is not something we can control experimentally; hence, here we designed a quasi-experimental study comparing MS in females with two control groups: males and females taking oral contraception. We compare MS across 2 days of the menstrual cycle where hormonal fluctuations peak: day 15 (corresponding with ovulation—peak estrogen) and day 22 (corresponding with mid-luteal phase—peak progesterone) based on self-reporting and physiological arousal via galvanic skin response (GSR). The inclusion of GSR as an objective measure of MS was to compensate for potential issues with the self-report measure, as outlined above, and to assess its potential correlation with subjective measures.

## Background

Motion sickness refers to the symptoms of malaise an individual experiences in response to different types of physical motion, such as traveling by car or boat (Money 1970). Common symptoms include nausea, vomiting, and sweating, and they can last anywhere from a few minutes to several days (Harm 1990). While traditionally associated with different modes of transportation, motion sickness has frequently been reported in cases where there is an absence of actual, physical motion (Golding and Gresty 2015), for example while viewing 3D videos (Solimini 2013), using mobiles and tablets (Stoffregen et al. 2014), playing console-based video games (Stoffregen et al. 2008) and using motionless simulators (Stoffregen et al. 2000). In the context of immersive VR, concern has grown regarding the prevalence of VR sickness (Cobb et al. 1999), as several studies have shown that it occurs even more frequently in VR than other forms of visual media (Sharples et al. 2008) and leads to high drop-out rates (Stanney et al. 2003).

No single effective intervention for preventing VR sickness exists and there is much debate regarding the causes of MS more generally. Previous research has suggested two main hypotheses: the toxin detector hypothesis suggests the brain has evolved to respond to incongruous sensory conflict which normally indicates that the body has ingested a toxin. To counteract this, the brain signals to the body to vomit (Treisman 1977). The vestibular-cardiovascular reflex hypothesis suggests that MS is the result of increased blood pressure due to cardiovascular reflex after stimulation of the vestibular system (Golding 2016). While both hypotheses can explain the resulting symptoms of MS, they fail to account for sex differences observed: they do not account for a wealth of research demonstrating that a significantly higher proportion of females experience motion sickness than males (Dobie et al. 2001; Flanagan et al. 2005; Koslucher et al. 2015; Lawson et al. 2004; Turner and Griffin 1999). For example, female participants score, on average, significantly higher than males on a Simulator Sickness Questionnaire (SSQ) after being exposed to a VR simulation (Chen et al. 2015).

Factors such as sex differences are poorly understood. With respect to qualitative data, sex differences may be influenced by a subjective reporting bias (Biocca 1992) where females tend to over-report symptoms while males under-report them so as to not appear ‘weak’ (Harm et al. 2007; Jaeger and Mourant 2001). This suggests that males and females may not actually differ in terms of their physiological experience of MS. However, this seems unlikely as there are several other animal species in which similar sex differences have been observed. For example, female shrews and rats are significantly more likely to vomit than males of the same species in response to motion stimuli (Javid and Naylor 1999; Zhou et al. 2017). Furthermore, there are MS studies with humans in which participants also drop-out due to vomiting (Stanney et al. 2003) and where significantly more female participants vomited in response to MS than males (Golding and Gresty 2015; Lawther and Griffin 1988). This cannot be explained by a reporting bias as vomiting is a reflexive physiological response. Sex differences may also be related to certain types of visual motion in which males typically have greater prior experience with using certain devices, which has allowed any symptoms they may have initially experienced to fade over time (Munafo et al. 2017). However, sex differences are evident even when using devices that males and females had been similarly exposed to in the past (Dobie et al. 2001).

This lack of understanding is a problem given the rise in popularity of home VR systems (Merhi et al. 2007). An insufficient understanding of the risk factors contributing to MS implies a failure to establish appropriate parameters for safe VR use (Stanney et al. 2003), which could potentially lead to legal, social, and economic repercussions (Calvert 2002; Kennedy et al. 2014; Swann and Stone 2002). Furthermore, VR is being increasingly applied outside of entertainment, such as in military training (Lele 2013), medical training (Aggarwal et al. 2006), astronaut training (Olbrich et al. 2018), and in some forms of psychotherapy (Kim et al. 2017). Women who experience significantly more VR sickness may, therefore, be at risk of receiving inadequate professional training in their careers, and this is especially concerning in domains such as science and the military where there are already deep-rooted gender inequalities (Kanny et al. 2014; Sjoberg and Via 2010). By addressing the underlying causes of sex differences in VR, we can develop ways of alleviating these symptoms to the benefit of female users. We are aware this is a contentious area of research (Lawson 2014; Saredakis et al. 2020), and hope to contribute to a better understanding of this issue with the findings we contribute here.

Understanding these sex differences from a physiological perspective may account for the shortcomings found in the literature. One theory proposes that they may be influenced by certain hormones that fluctuate throughout the menstrual cycle (Cheung et al. 2001; Golding et al. 2005; Matchock et al. 2008). However, research in this area is still relatively sparse and has so far produced inconsistent results. For instance, previous research suggests that females experienced significantly more MS on day 5 during the menstrual phase of their cycle when traveling by boat (Golding and Gresty 2015), while others suggest that they experience significantly more MS during the peri-menstrual phase of their cycle, compared to other days, during exposure to a rotating optokinetic drum (Matchock et al. 2008). In contrast, other research is inconclusive suggesting no effect of the menstrual cycle on MS at all when induced by Coriolis or exposure to a visual display unit (Cheung et al. 2001; Howarth and Clemes 2006). However, the stimuli and symptom measurement methods used throughout the literature are inconsistent, and all define and measure menstrual phases differently (Golding 2016) which may explain why results are mixed. For example, some broadly defined the cycle in terms of peri-menstrual (days 25–10) and peri-ovulatory (days 11–24) phases which allowed for a large variation in participation (Matchock et al. 2008), while others defined it in terms of menstrual (day 5), ovulatory (day 12), mid-luteal (day 19), and premenstrual (day 26) phases which is far more narrow and specific (Golding et al. 2005; Howarth and Clemes 2006).

In the context of VR sickness, research concerning the influence of the menstrual cycle is even less common with only one existing study to date, perhaps reflecting the difficulty of this research. Previous research has investigated how VR sickness varied across days 5, 12, 19, and 26 of the menstrual cycle, during which estrogen and progesterone fluctuate, in 16 naturally cycling females, 16 females using a combined monophasic contraceptive pill, and 16 males (Clemes and Howarth 2005). The contraceptive group was included as a control as the combined pill maintains constant rather than fluctuating hormone levels, and therefore specific hormone effects are isolated to the non-contraceptive female group. Hormone levels were objectively confirmed via the extraction and analysis of saliva samples. Participants were tasked with playing a fast-paced video game while wearing a HMD. For all female participants, the order of testing was counterbalanced to avoid primacy or recency effects, in which four groups of four females in each group began on a different testing day. Symptoms were assessed via 1-min interval malaise ratings on a scale from zero to four, a VR sickness symptoms questionnaire administered pre- and post-immersion, and symptom onset time (i.e., the first time a participant reported an increase in malaise on the four-point scale).

Compared to males and females using contraception, symptoms were significantly worse for naturally cycling females only on day 12 of their menstrual cycle, during which estrogen levels begin to rise. No significant differences in VR sickness across the 4 days of testing were found for either the oral contraceptive or the male group. These preliminary findings by Clemes and Howarth suggest that females may be more susceptible to VR sickness because fluctuating levels of estrogen during the ovulatory phase of their cycle may modulate this. Finally, all other previous studies have produced conflicting results, defined menstrual cycle phases inconsistently, and did not investigate VR specifically, making it difficult to draw any reliable conclusions from their results regarding the effect of hormones on MS in females using VR. Moreover, the potential role of adaptation over time as a factor impacting VR sickness over time has not been evaluated independently in this work.

### Study structure

To address these issues, we investigated if MS in VR is related to hormonal variations that occur throughout the menstrual cycle. Participants were assigned to groups based on their self-reported menstrual cycle phase and tested on day 15 (ovulation—peak estrogen) and day 22 (mid-luteal phase—peak progesterone) of the menstrual cycle Our rationale was based on more recent research delineating the typical hormone profiles observed in the average cycle (Allen et al. 2016) and their effects on physiology and neurology, e.g., dysmenorrhea (Bernardi et al. 2017) and migraines (Borsook et al. 2014; Brandes 2006), and how this might be linked to the experience of VR sickness. Furthermore, higher levels of estrogen and progesterone are known to induce gastric dysrhythmia in women (Walsh et al. 1996) which may be linked to the experience of nausea and vomiting (Koch 2002). One challenge here is that the exposure to VR coincided with the order in which the cycles were experienced, and we note this impact on the conclusions in the Discussion.

VR sickness was measured in four different ways to provide a multi-dimensional assessment of symptoms. The first (Immersion Duration) was a behavioral measure which assessed how long participants could play the VR game before having to stop due to feeling motion sick. The second was a retrospective self-reported measure using a questionnaire, the Simulator Sickness Questionnaire (SSQ) (Kennedy et al. 1993), considered a robust instrument to assess subjective experiences of VR sickness (Balk et al. 2013). It contains a list of 16 symptoms which participants rate themselves experiencing as either “none”, “slight”, “moderate”, or “severe”. These are given a numeric value and combined into an overall VR sickness score, and symptoms can further be divided into three sub-groups representing three general dimensions of VR sickness including Nausea, Disorientation, and Oculomotor Discomfort. The third measure (Discomfort) was a self-reported measure which had participants rate their discomfort on a scale from zero to ten throughout the VR game. We used a discomfort score on top of the SSQ because it is a self-report method which allows us to evaluate participants’ subjective experiences of VR sickness directly during the game, whereas the SSQ is administered after the game, and therefore requires participants to evaluate their experience retrospectively. Finally, VR sickness was physiologically assessed using a BioPac which measures each participant’s galvanic skin response (GSR), or sweat conductance, via electrodes attached at the fingertips of one hand. This method gives an indication of participants’ degree of physiological arousal and was chosen because high levels of physiological arousal are associated with higher VR sickness severity (Whalen et al. 2003; Yokota et al. 2005). Furthermore, it is not subject to a reporting bias (Min et al. 2004). Our motivation for using a combination of both subjective and objective measurements of VR sickness was to assess whether a unified objective and subjective overall profile of each participant’s experience of VR sickness is possible with the measures used here.

Based on prior work (Clemes and Howarth 2005), we hypothesized that: first, there will be significant differences for measures of VR sickness and discomfort when playing a VR game between males and naturally menstruating females on day 15 but not on day 22 due to higher levels of estrogen on day 15 or adaptation over time across a longer period than previously known (Hill and Howarth 2000). Second, similarly there will be significant differences for measures of VR sickness between females using contraception and naturally menstruating females on day 15 but not on day 22.

### Participants

Participants were recruited through research advertisement posters, an online research participation scheme (RPS), online university notice-boards and social media. Exclusion criteria included anyone with self-reported high light sensitivity, epilepsy, or non-normal/normal-to-corrected vision, females who reported themselves as having highly irregular menstrual cycles, and females using any oral or surgical forms of contraception (e.g., contraceptive implant, IUD) other than the combined monophasic pill. As male participants do not experience menstruation, they were assigned a “pseudo-cycle” and informed that they were to participate on days 15 and 22 of this cycle. Female participants were assigned to either a “natural menstruation” or “oral contraception” group depending on whether or not they were currently taking a combined monophasic contraceptive pill.

A total of 30 participants, including 10 males and 20 females, were recruited. They made up our three participant groups such that there were ten females in the natural menstruation group with a mean age of 23 (SD = 5.42), ten females in the oral contraception group with a mean age of 21.20 (SD = 2.82), and ten males in the male group with a mean age of 22.20 (SD = 1.99). Based on the large effect sizes reported for malaise and nausea in prior work (e.g., 2.18 in Clemes and Howarth 2005), a power analysis using G*Power 3.1.9.4 (Faul et al. 2007) for the ANOVA interactions presented below required a sample size of nine participants per group.

### Game experience and study apparatus

VR sickness was induced using a custom-designed sledding-based VR game. This was done to eliminate the effects of any prior experience participants may have had which could have influenced VR sickness severity had we used a commercially available VR game, as VR sickness is shown to significantly decrease with repeated exposure to the same virtual environment (VE) (Keshavarz 2016).

Operating a virtual snow sleigh, the goal of the game was to reach the end of a predetermined sledding course while avoiding crashing into snowmen obstacles randomly placed within three implied lanes of the course. Players moved automatically forward through the course similar to moving on the rails of a rollercoaster: this was to encourage participants to move in the game as much as possible to avoid the unpleasant sensation of colliding with a snowman, thus ensuring that they all moved a similar amount, instead of letting them explore the VE at their own pace. The course traversed a flat, mountain environment consisting of a series of straight sections and bends around which the player was automatically turned, guaranteeing some movement to induce VR sickness. The game was played using a head mounted display and from a first-person perspective, placing players directly in the driving seat of the sleigh. Players controlled the game avatar by tilting their head left and right allowing them to drift between the lanes.

**Fig. 1:**
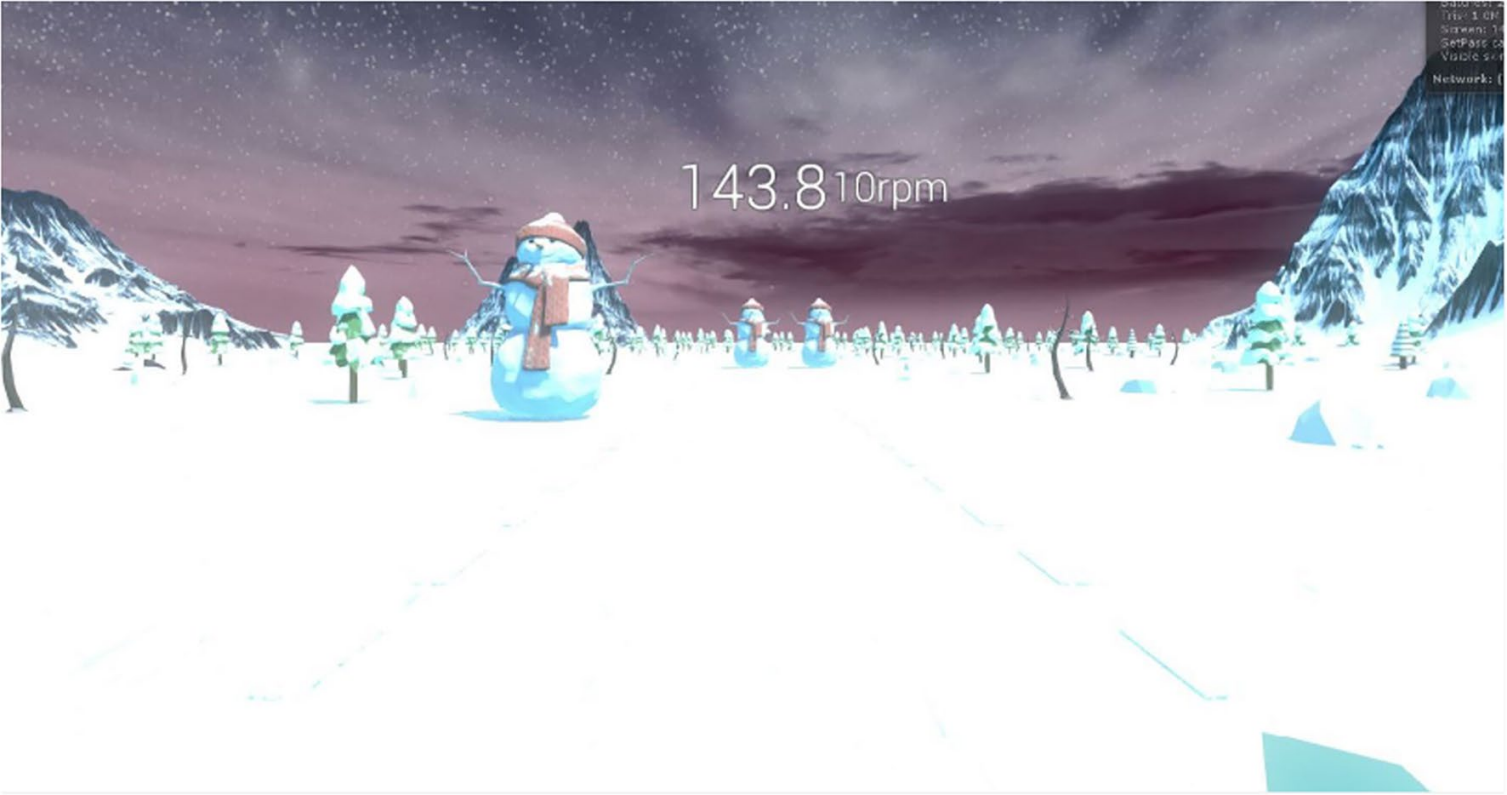
VR gameplay from the player’s perspective in the middle lane of the course facing the snowmen obstacles

**Fig. 2:**
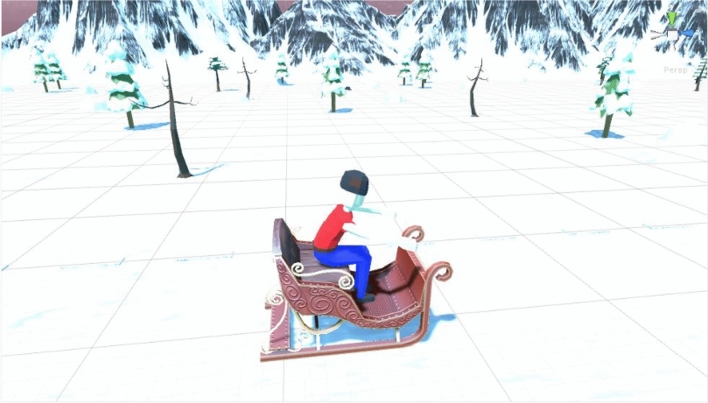
Game avatar from an outsider perspective, stationary and prior to game-start

Participants remained seated throughout the game to keep their body still and confine movement to the head. This was to ensure that any VR sickness experience resulted from the visuals of the game rather than the actual, physical motion of the participant’s body. 3D models were fully textured in the environment, complete with a skybox to emphasize the apparent motion of the user through the environment. The game consisted of players seated in a snow sled, upon which they move through the game as if on a roller coaster: this design is referred to as ‘on rails’ as players have limited movement to the left and right but may not move forward and/or influence the speed at which they are moving. Participants also wore headphones through which game music and sound effects (e.g., colliding with a snowman) were played. This was to enhance the feeling of colliding with snowman obstacles and to make the game more immersive. Examples of the VR gameplay and game avatar can be seen in Figs. 1 and 2.

**Fig. 3:**
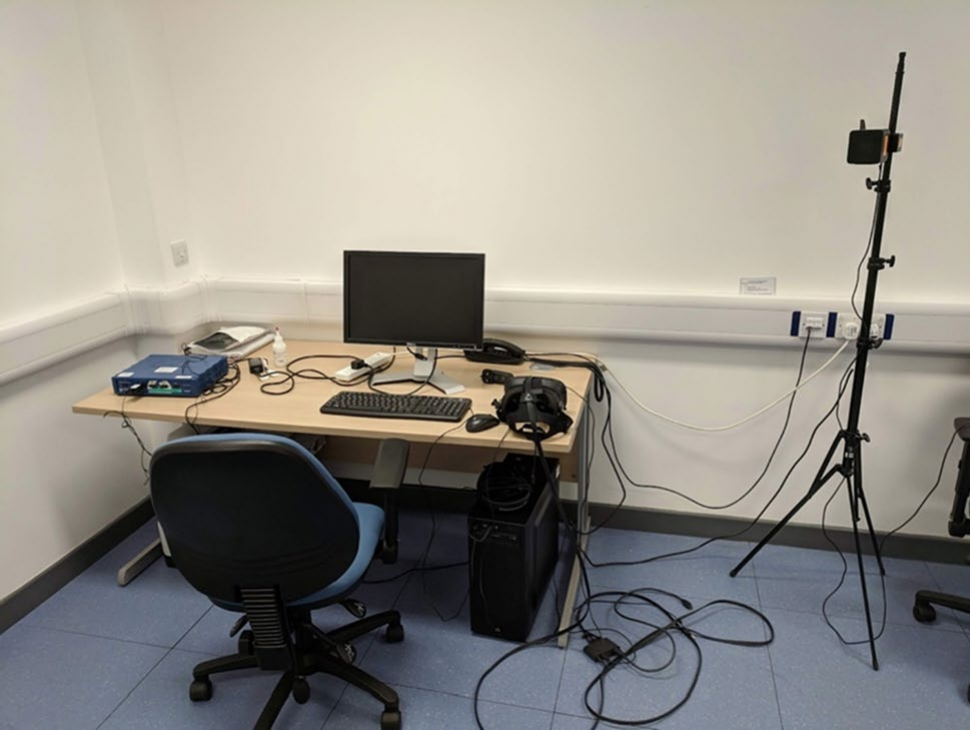
Laboratory set-up with HTC Vive headset (right-hand side of desk) and front base station (far right of image); the rear base station (not pictured) was placed on the left behind the desk chair facing both the desk and front base station

The game was presented using a HTC Vive headset connected to a desktop computer running on a Windows 10 operating system and a GeForce GTX 980 graphics processing unit. It was connected via HDMI and USB 2.0 cables with head motion tracked via two HTC Vive base-stations, as can be seen in Fig. 3, which translated real motion into movements within the game, such that a leftward head-tilt produced a leftward movement of the game avatar. The HTC Vive has a better resolution, a wider field of view (FoV), and weighs nearly half the amount of the models used in previous studies (Clemes and Howarth 2005). This allowed us to control for the confounding effects of weight, FoV, and other factors of the HMD which are known to induce VR sickness (Al Zayer et al. 2019).

### Design and procedure

This study had a mixed design in which the three independent quasi-experimental groups were subjected to repeated-measures as they were all tested on day 15 and on day 22 of the menstrual cycle. The independent variable (IV) was day of cycle/pseudo-cycle, and the dependent variable (DV) was motion sickness measured four ways to provide a multi-dimensional assessment of symptoms: a behavioral measure (Immersion Duration), a retrospective self-reported measure (SSQ), a self-reported measure (Discomfort score), and a physiological measure (GSR). It is important to note here that *all* participants were tested on day 15 *first*, with no counterbalancing for day 22. The rationale for this is that we minimize the risk of other confounding factors influencing individuals by maintaining a shorter window between tests. Adaptation—in our case, the relief of VR symptoms—may very well manifest through time spent outside VR, but this may happen at different times for different people, and would be unrelated to the hormonal nature of the menstrual cycle, the basis of our hypotheses. By testing some participants on day 22 first, these participants would have had a great individual temporal window between tests, in turn increasing the uncertainty around whether adaptation had occurred. Simply put, it is unknown how this longer period may have interfered with individuals’ adaptation response to VR. Thus, we made the decision to keep testing schedule constant.

Prior to immersion, participants were given an information sheet followed by a consent form to sign, and then completed an online pre-immersion SSQ to gather a baseline assessment of their symptoms. Following this, they were fitted with the VR headset and the experimenter explained the principles of the game. GSR was measured before the game for 3 min to get a baseline value, and continued to be measured throughout the duration of the game.

The game lasted a maximum of 20 min. This time was selected because a longer duration may have confounded the results, as long exposure significantly influences chances of experiencing motion sickness (Ruddle 2004; Stanney et al. 2003), and to avoid any serious side effects. Throughout, participants were asked to rate their discomfort levels on a scale from zero to ten at four 5-min intervals (5 min, 10 min, 15 min, 20 min) when verbally prompted to do so by the researcher. If a participant stopped before the 20 min was up, any subsequent 5-min interval discomfort scores they had not yet been able to complete was assumed to be at the maximum score of 10. Participants were encouraged to stop as soon as they felt that symptoms became too uncomfortable to bear, and their immersion duration was recorded. The headset and GSR electrodes were then removed and participants completed a post-immersion SSQ.

As this study has both an independent-samples and a repeated-measures IV, the data were then analyzed in JASP using mixed analyses of variance (ANOVA) for each measure of VR sickness. Pre–post change scores were calculated by subtracting the pre-immersion value from the post-immersion value for the SSQ measure and by taking the mean of scores across time for the discomfort score and GSR measures as these were assessed at multiple continuous time-points. All figures were plotted using ggplot in R (v4.3), specifically using the functions stat_halfeye, geom_boxplot, and ggdist::stat_dots to provide visualizations of all the data points and distributions.

## Results

### Immersion Duration

**Fig. 4:**
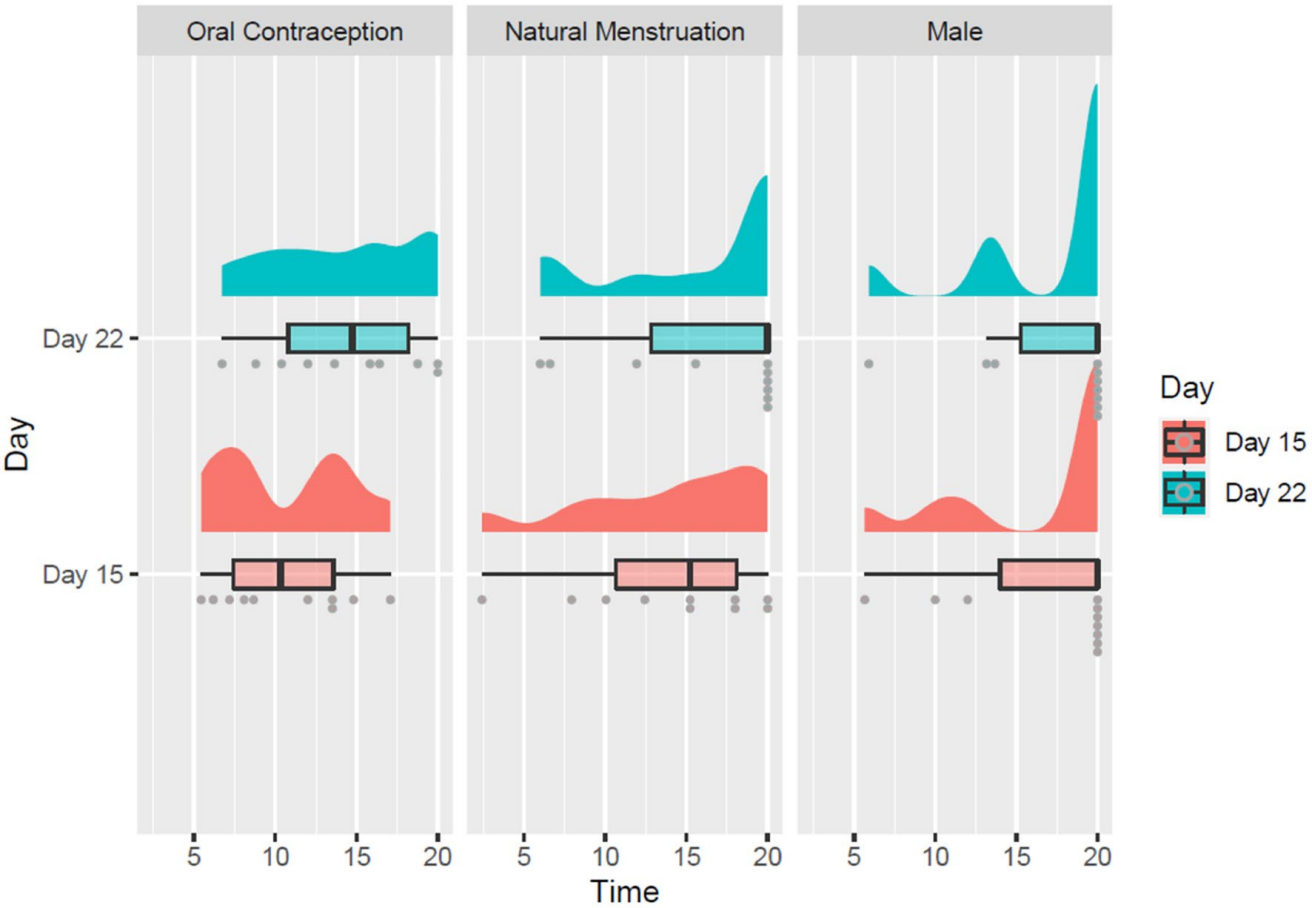
Immersion Duration (time) in minutes by group (oral contraception female, natural menstruation female, male) and by day of testing (15, 22), depicted with rain cloud plots and box plots

There was a significant main effect of day of cycle on immersion duration times overall, *F* (1, 27) = 7.84, *p* = 0.009, *η*^*2*^ = 0.037, with participants able to remain immersed in the VE for significantly longer on day 22 than on day 15 regardless of group. There was no significant main effect of group on immersion duration times overall, *F* (2, 27) = 2.36, *p* = 0.114, *η*^*2*^ = 0.122, and no significant interaction between day of cycle and group in terms of immersion duration times, *F* (2, 27) = 1.47, *p* = 0.248, *η*^*2*^ = 0.014. An illustration of these results can be seen in Fig. 4.

### SSQ scores

There was no significant main effect of day of cycle on SSQ scores (see Table 1) overall, *F* (1, 27) = 2.56, *p* = 0.121, *η*^*2*^ = 0.011, and no significant main effect of group on SSQ scores overall, *F* (2, 27) = 0.623, *p* = 0.544, *η*^*2*^ = 0.039. There was also no significant interaction between day of cycle and group in terms of SSQ scores, *F* (2, 27) = 0.519, *p* = 0.601, *η*^*2*^ = 0.004.

**Table 1:** SSQ scores by group and day of cycle

Group	Pre 15	Post 15	Pre 22	Post 22
Oral contraception	3.1	16.1	4.6	17.2
Natural menstruation	3.8	19.2	4.7	17.0
Male	3.3	14.1	3.2	12.3

### Discomfort scores

**Fig. 5:**
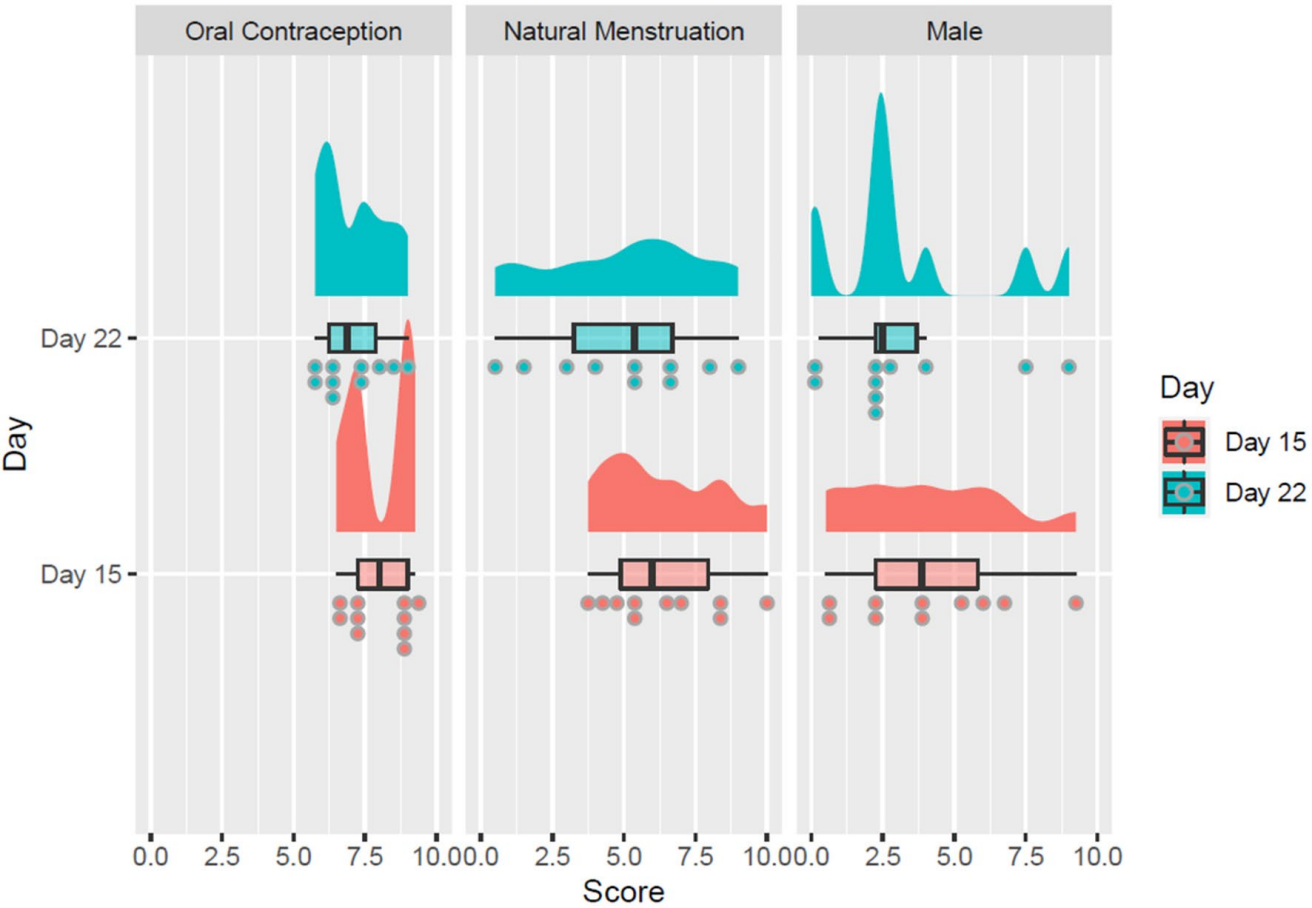
Average discomfort score by group (oral contraception female, natural menstruation female, male) and by day of testing (15, 22), depicted with rain cloud plots and box plots

With respect to H1, there was a significant main effect of day of cycle on discomfort scores overall, *F* (1, 27) = 10.85, *p* = 0.003, *η*^*2*^ = 0.036, with participants reporting significantly higher discomfort on day 15 than on day 22 regardless of group, as illustrated in Fig. 5. There was also a significant main effect of group on discomfort scores overall, *F* (2, 27) = 8.577, *p* = 0.001, *η*^*2*^ = 0.339. To identify the nature of this effect, post hoc contrasts were conducted using Bonferroni-corrected *t *tests. They indicated that the mean discomfort scores of the oral contraception group (*μ* = 7.54, *σ* = 1.12) were significantly higher than the mean discomfort scores of the male group (*μ* = 3.69, *σ* = 2.83),* p* = 0.001. However, mean discomfort scores did not significantly differ between the oral contraception and the natural menstruation (*μ* = 5.69, *σ* = 2.39) groups (*p* = 0.17) or between the male and the natural menstruation groups (*p* = 0.12). There was no significant interaction between day of cycle and group in terms of discomfort scores, *F* (2, 27) = 0.335, *p* = 0.718, *η*^*2*^ = 0.002.

### GSR

**Fig. 6:**
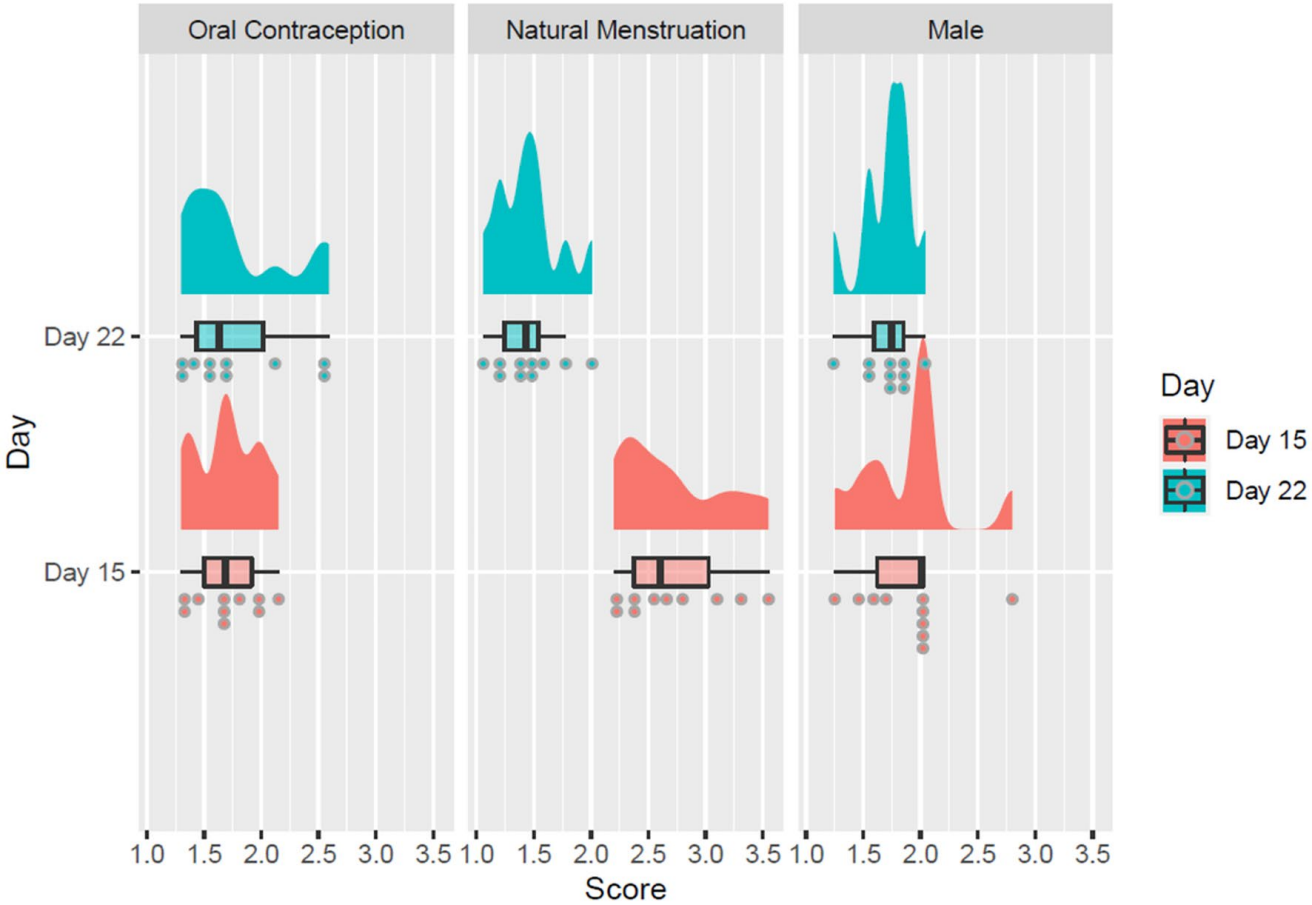
Average levels of physiological arousal in microsiemens (μS) by group (oral contraception female, natural menstruation female, male) and by day of testing (15, 22), depicted with rain cloud plots and box plots

There was a significant main effect of day of cycle on physiological arousal overall, *F* (1, 27) = 51.22, *p* < 0.001, *η*^*2*^ = 0.184 with participants experiencing significantly higher levels of physiological arousal on day 15 than on day 22 regardless of group, but there was no significant main effect of group on physiological arousal overall, *F* (2, 27) = 3.159, *p* = 0.059, *η*^*2*^ = 0.08. However, there was a significant interaction between day of cycle and group in terms of physiological arousal, *F* (2, 27) = 41.44, *p* < 0.001, *η*^*2*^ = 0.297. Post hoc contrasts were conducted using Bonferroni-corrected paired samples *t *tests, indicating that the mean physiological arousal levels differed significantly between day 15 (*μ* = 2.72, *σ* = 0.46) and day 22 (*μ* = 1.45, *σ* = 0.28) for the natural menstruation group, *t*(9) = 9.26, *p* < 0.001, with participants from this group experiencing significantly higher levels of arousal on day 15 as illustrated in Fig. 6. The mean physiological arousal levels did not significantly differ between day 15 (*μ* = 1.71, *σ* = 0.28) and day 22 (*μ* = 1.78, *σ* = 0.47) for the oral contraception group (*p* = 0.44), nor between day 15 (*μ* = 1.89, *σ* = 0.43) and day 22 (*μ* = 1.72, *σ* = 0.22) for the male group (*p* = 0.12).

## Discussion

All participants reported similar levels of VR sickness in the SSQ across both phases of the menstrual cycle, although on average they demonstrated higher levels of physiological arousal on day 15, their first exposure to the VR game, regardless of the group they were in. Potentially consistent with both H1 and H2, females in the natural menstruation group experienced significantly higher levels of physiological arousal during ovulation (day 15) compared to males and compared to females in the oral contraception group. This finding could be due primarily to menstruation interacting with VR sickness; however, there could be other causes. These results could be explained by oral contraceptive side effects: indeed, the high levels of estrogen and progestin contained in the combined pill can cause some users to experience headaches and nausea (Frye 2006; Teepker et al. 2011) which also commonly occur with VR sickness. It is possible that participants in the oral contraception group were experiencing these side effects during the study, which would have caused them to report higher discomfort during both phases of the menstrual cycle.

A greater concern is that this physiological data contradicts the pattern in subjective reports, because there were no significant differences found with the SSQ data as the primary measure of VR sickness. Yet, according to discomfort scores and immersion duration, all groups experienced a higher degree of VR sickness on day 15 (during ovulation for the natural menstruation group) and a lower degree of VR sickness on day 22. This contradiction could mean that naturally menstruating females under-reported how much VR sickness they were experiencing if the GSR results were an objective measure of VR sickness; the fact that the pattern is not consistent for all groups draws this conclusion into question. Note that GSR has been found to increase at the ovulatory stage, and so indeed might not provide a clear measure of just VR sickness (Gómez-Amor et al. 1990). Moreover, some prior literature found the opposite (Biocca 1992), that female participants over-reported their symptoms and male participants under-reported their symptoms. In addition, memories of painful or uncomfortable sensory experiences are known to be susceptible to bias (Redelmeier and Kahneman 1996).

Regardless of the group they were in, on average all participants were able to remain immersed in the VR game for longer on day 22 (during the mid-luteal phase—progesterone peak) than day 15 (during ovulation—estrogen peak). These results could be explained by a remarkable adaptation effect or competitiveness, and perhaps contradictory to H1 and H2. First, in terms of adaptation, it is likely that having repeated the experience made the participants feel less likely to be disturbed by it the next time it was experienced. Discomfort was lower in all groups on day 22, and a sensible solution might be to find ways to encourage participants to attempt a VR experience more than once to lessen their discomfort. One such way might be through competition. Indeed, as participants were informed that the goal of the game was to avoid colliding with snowmen obstacles, they could have been motivated by a sense of competitiveness to play and remain immersed for longer (Vorderer et al. 2003). Unfortunately, we did not record a performance score or prior experience with gaming due to our focus on physiological, not social, factors in VR sickness, but such a measure might be informative in future research. They may have wanted to try and improve their performance during the mid-luteal phase compared to during ovulation, which may explain why they all remained immersed for longer during the mid-luteal phase. However, as competitiveness was not measured, we cannot know for sure. Adaptation does not necessarily rule out the role of estrogen levels; if contraceptives maintain near constant hormone levels, then the lack of a change in variance for oral contraception compared to natural menstruation group would imply that adaptation is not a sole contributing factor. Future research should aim to investigate and control for competitiveness as a potential factor influencing behavioral measures of VR sickness, and a means of encouraging a repeat experience if indeed adaptation is the key to lowering discomfort.

Our design also considered the impact of repeated exposure on VR sickness. It is possible that exposure at days 15 and 22 could result in an increase in discomfort (and decrease in duration), or instead a decrease in discomfort (and increase in duration). It appears the former possibility is the case: our participants seem to have experienced a certain degree of adaptation during the week that elapsed between day 15 (ovulation) and day 22 (the mid-luteal phase), in which previous exposure to the game was sufficient to significantly reduce the amount of VR sickness they experienced in the second exposure session (Hill and Howarth 2000). Indeed, across all participants, there was a simultaneous decrease in discomfort and increase in immersion duration between ovulation and the mid-luteal phase. Interestingly, this may indicate that habituation to virtual motion can occur with less-frequent exposure than previous research has suggested. Howarth and Hodder (2008) argue that a delay lasting longer than 2 days between VR exposure sessions will not be enough to enable habituation, whereas in our study, there was a delay of 7 days. It is unclear whether habitation to an environment or habituation to the experience of virtual reality more generally is influencing discomfort scores; therefore, one worthwhile method to combat VR sickness may be repeated exposure to the same environment. However, this also means that we cannot test whether the results we observed are necessarily due to a difference in hormone levels between day 15 and day 22 of the menstrual cycle. Future research should counterbalance the number of participants exposed to the VR game for the first time on day 15 with a similar amount being exposed for the first time on day 22. If it remains the case that there is habituation with repeated exposure, then this might suggest a potential treatment approach as a solution. Note, however, that although discomfort might not improve, the length of time a participant can withstand it increases, so it may not be an ideal solution.

Compared to previous research on VR sickness, the current study presents some notable differences. The closest prior work to our own (Clemes and Howarth 2005) is distinct such that their study involved a game played with a controller and participants’ heads were held stable with a chin rest—an atypical combination for state-of-the-art interaction in the context of immersive virtual reality—making it a poor comparison. Second, the phases of the menstrual cycle chosen as the focus of the study were defined based on more recent research on menstrual hormone profiles, as well as research linking the effects of estrogen and progesterone to nausea, migraines, headaches, and gastric dysrhythmia. Finally, discomfort was measured at 5-min rather than 1-min intervals to reduce external distractions and increase participants sense of presence in the VE, as increased sense of presence has been found to positively correlate with increased VR sickness (Hettinger and Riccio 1992; Lin et al. 2002). Measuring discomfort required participants to be tapped on the shoulder and to interact with the researcher briefly verbally, which could have been disruptive to their overall sense of presence at 1-min intervals. Alternative methods may involve experience sampling (Barathi et al. 2020) to mitigate disruption and maintain sense of presence within the VE.

More research is needed to identify the specific role hormones play in VR sickness independent of the potential adaptive effects of multiple exposures, such as their potential to trigger nausea-like symptoms within the gastro-intestinal system. This could be done for instance using ingestible gastro-intestinal sampling devices (Amoako-Tuffour et al. 2014). Crucially, these results indicate the need for future research to determine whether VR developers should create safety guidelines for female users, for instance by advising them that they may experience significantly more VR sickness as a function of the menstrual period, or by seeking other ways to ensure that VR systems are built to be inclusive, safe, and accessible to all users, mitigating the risk of losing out on a large consumer base. For example, other aspects of distortions in the display and latency issues in relating movement to visual perception might be at the root of MS in VR, and solving those issues might mitigate the risk of VR sickness.

## Limitations and future work

While high levels of physiological arousal can be associated with higher degrees of VR sickness, they may also indicate excitement (Kucher et al. 2016), so it is possible that the high levels of arousal found in the current study may also partly be due to participants finding the game exciting. Furthermore, oral contraceptives may alter body fluid regulation such as sweating (Stachenfeld et al. 1999), which is what GSR measures. Some may argue a more appropriate physiological measure would be something like gastric dysrhythmia, which is much more specific to nausea, or heart rate variability—though that too is multiply determined. We also did not have a pre-immersion measure of symptoms other than self-report (SSQ), and the session lasted only 20 min; having a longer period of assessment would be important for future research particularly as VR might become a feature of work in the near future and worn for longer periods of time. Furthermore, while some research suggests that females are quite accurate at estimating specific days and phases within their menstrual cycle (Creinin et al. 2004), this can depend on the average length of their cycle (Small et al. 2007) and may be negatively impacted by those who have been using oral contraceptives for a long time. Furthermore, hormone levels throughout the menstrual cycle may fluctuate daily and between cycles. It is, therefore, possible that some of our participants did not participate on the correct days. Future research should attempt to replicate the current study using objective measures of hormone levels.

Though randomization mitigates some unsystematic variation in an experiment, and our quasi-experimental design contains some limitations, we found an overall significant effect of group—naturally menstruating (experimental), oral contraceptive (control), and male (control)—on VR sickness and that adaptation may occur with even a week between exposures. Yet, there are other factors that can influence VR sickness worth considering in future studies. For instance, studies should control for whether participants have eaten before taking part, as research shows that eating can actually decrease the degree of MS experienced by altering activity in the autonomic nervous system (Muth 2006). Finally, future studies could control for participants’ prior gaming experience as previous exposure to VR may affect subsequent experiences of VR sickness (Häkkinen et al. 2006). Previous studies have also suggested controlling for previous exposure to other gaming platforms (Häkkinen et al. 2002); however, other research has argued that these are qualitatively different from VR and that MS habituation is, therefore, not likely to occur across platforms (Munafo et al. 2017). Future research could, therefore, also aim to replicate the present study while ensuring that participants have not eaten beforehand and have had similar prior exposure to VR devices specifically (Brown et al. 2020). Of course, no single study will definitively settle the issue of sex differences in VR sickness (Lawson 2014; Saredakis et al. 2020), but this work provides more data to begin to clarify the issue.

## Data Availability

On request.
